# Redefining sensorimotor mismatch selectivity in the visual cortex

**DOI:** 10.1016/j.celrep.2023.112098

**Published:** 2023-02-22

**Authors:** Tomaso Muzzu, Aman B. Saleem

**Affiliations:** 1 UCL Institute of Behavioural Neuroscience, Department of Experimental Psychology, University College London, 26 Bedford Way, London WC1H 0AP, UK

**Keywords:** predictive coding, mouse vision, locomotion

## Abstract

This Matters Arising Response contains our commentary to the response written by Vasilevskaya et al., 2023, publishing concurrently in *Cell Reports*, for our recent article “Feature selectivity can explain mismatch signals in mouse visual cortex.” We find that results in the response reinforced many of our findings and, further supported by their new results, we argue for the necessity to redefine sensorimotor mismatch selectivity in the mouse visual system.

## Highlights

• Visual flow perturbation responses in open-loop can be larger when animals run
• Sensorimotor mismatch selectivity should be redefined accounting for these responses
• Previous results on sensorimotor mismatch need to be interpreted with caution

## Teaser

In this Matters Arising Response, Muzzu and Saleem argue that the new results of the Matters Arising by Vasilevkaya et al. support many of their original findings. They reiterate the need to redefine sensorimotor mismatch selectivity in the mouse visual system.

## Main text

**Figure 1:**
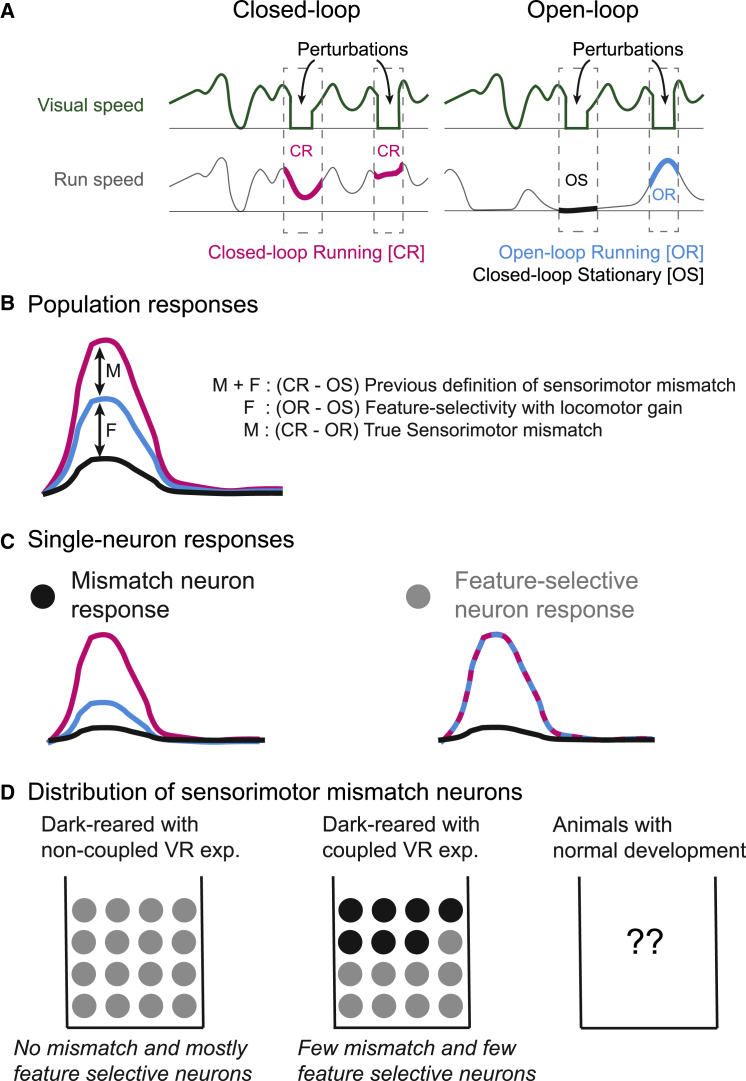
Differentiating mismatch and visual feature selectivities (A) Illustration of the stimulus conditions used in the studies, when perturbations of the visual stimuli occur when the animal is in closed-loop (CR) or when they occur when the same perturbation stimuli is replayed to the animal in open-loop when the animal is stationary (OS) or running (OR). (B) Schematic of population response to visual perturbation in closed-loop (CR, magenta), open-loop running (OR, blue), and open-loop stationary (OS, black) conditions. We show that the response “F” (OR vs. OS) can be explained by feature selectivity. Previous work used “M + F” (CR vs. OS) to define sensorimotor mismatch responses in the visual cortex. We propose “M” (CR vs. OR) as the true definition of mismatch selectivity. (C) Schematic representations of responses of a true mismatch neuron, which shows a difference in response between perturbations during OR and CR, and feature-selective neurons, which do not differentiate the same conditions. (D) Expected distribution of previously defined mismatch (M + F) neurons. Animals that were dark reared and only experienced closed-loop conditions (motor-coupled virtual reality) showed OR responses that were intermediate between OS and CR. Therefore, we can expect some of the previously defined mismatch neurons to be feature-selective neurons and some to be true mismatch neurons (center). On the other hand, dark-reared animals that only experienced non-coupled virtual reality do not show differences between OR and CR and therefore are likely to have only feature-selective neurons (left). Whether there are any mismatch neurons or what fraction of neurons are present in animals raised under normal lab conditions is still unknown (right).

Sensorimotor mismatch selectivity was previously reported based on a definition as the difference between the responses to visual flow perturbation in closed-loop in running animals (CR) and the response to the same stimuli replayed when animals are stationary, open-loop stationary (OS). In our article,^5^ we showed that perturbations of visual flow can elicit significantly stronger responses in open-loop when animals are running (OR) compared with when OS. We found that OR responses can be explained by the selectivity of cells to certain visual features (F), combined with locomotion-induced gain, an established phenomenon in the mouse visual system.^1^^,^^2^^,^^3^^,^^4^ However, we did not directly compare CR with OR responses (M), which we suggested as a more rigorous proof of sensorimotor mismatch selectivity. Previous work, however, used a mixture of the above two signals “M + F” (CR vs. OS) to define sensorimotor mismatch responses in the visual cortex. Based on the results from our study^5^ and the Matters Arising article,^6^ we propose a more nuanced definition of sensorimotor mismatch selectivity (CR vs. OR) to account for the visual feature selectivity of neurons (Figure 1).

Below, we discuss sensorimotor mismatch responses at two scales, population activity and of individual neurons, as we feel the two approaches reflect the different perspectives on the interpretation of our results.

### Mismatch selectivity in population responses

Vasilevskaya et al. (2023)^6^ re-analyzed data from two prior studies,^7^^,^^8^ collected from animals with particular developmental experiences. Specifically, one group of animals was reared in the dark with six 2-h-long experiences of closed-loop virtual reality. In these animals, they found that the population responses to CR visual flow perturbations were greater than those to OR. This is indeed a convincing demonstration of mismatch responses in population activity. However, we would like to also highlight that in the second group of animals, which were also dark reared and, instead, experienced open-loop virtual reality, the responses to CR and OR were comparable. Yet, since the population response to CR is greater than OS, the original definition of sensorimotor mismatch selectivity would wrongly conclude the existence of mismatch responses (albeit smaller) in the population.

Therefore, while CR-experienced animals can show mismatch selectivity, and we appreciate that this is the first test of “true” sensorimotor mismatch responses, these new results also highlight two features. Firstly, it is crucial to use a more stringent definition of mismatch selectivity as locomotion-enhanced feature selectivity might wrongly be considered mismatch selectivity. Secondly, experience can greatly influence responses, so it is important to reassess mismatch selectivity, as defined here, in animals with a normal developmental experience.

### Mismatch-selective neurons

A major difference in perspective between the two articles lies in the definition of mismatch selectivity that is based on either population or single-cell responses. In our study, we found that neurons that have larger positive responses to visual perturbations tend to be more selective to low temporal frequencies. Characterizing responses of individual neurons allows for the essential juxtaposition of selectivity of visual features to the new features observed.

To date, individual mismatch-selective neurons have only been characterized based on CR versus OS, and the article by Vasilevskaya et al. (2023)^6^ did not characterize neurons with respect to their CR versus OR responses or to their visual feature selectivity properties (Figure 1C). Therefore, we renew our initial suggestion: “feature selectivity can explain mismatch signals” in many individual neurons if their selectivity is exclusively tested based on the CR versus OR responses.

Another important factor to consider is the incidence of mismatch-selective neurons in a population. Previous reports quantified mismatch-selective neurons to be around 10% of the active population based on a definition of CR versus OS.^9^^,^^10^ Considering that some of these would be feature-selective neurons, redefining neural responses with a more precise criterion (CR vs. OR) will yield a more accurate, and likely smaller, count of mismatch-selective neurons (Figure 1D).

### Conclusion

The new results show that responses to visual perturbations when an animal is running in open-loop are intermediate to when the animal is stationary and when the animal is running in closed-loop. Therefore, previous results based on the definition of mismatch selectivity as closed-loop running versus open-loop stationary need to be interpreted with caution as there remains the possibility that some effects are explained away by simple locomotion-induced gain of feature selectivity, rather than sensorimotor mismatch. Going forward, it is imperative to use a definition of mismatch selectivity that is beyond locomotion-induced gain of low temporal frequency tuned neurons, and thus redefine mismatch responses as the difference in responses between the running conditions of closed-loop and open-loop.
